# Transcriptomic response to aquaculture intensification in Nile tilapia

**DOI:** 10.1111/eva.12830

**Published:** 2019-07-17

**Authors:** Deiene Rodriguez‐Barreto, Olivier Rey, Tamsyn M. Uren‐Webster, Giovanni Castaldo, Sonia Consuegra, Carlos Garcia de Leaniz

**Affiliations:** ^1^ Centre for Sustainable Aquatic Research (CSAR), College of Science Swansea University Swansea UK; ^2^ Université de Perpignan Via Domitia Perpignan France; ^3^ Systemic Physiological and Ecotoxicological Research, Department of Biology University of Antwerp Antwerp Belgium

**Keywords:** aggression, aquaculture intensification, crowding, fish domestication, gene expression, HPI axis, stress response

## Abstract

To meet future global demand for fish protein, more fish will need to be farmed using fewer resources, and this will require the selection of nonaggressive individuals that perform well at high densities. Yet, the genetic changes underlying loss of aggression and adaptation to crowding during aquaculture intensification are largely unknown. We examined the transcriptomic response to aggression and crowding in Nile tilapia, one of the oldest and most widespread farmed fish, whose social structure shifts from social hierarchies to shoaling with increasing density. A mirror test was used to quantify aggression and skin darkening (a proxy for stress) of fish reared at low and high densities, and gene expression in the hypothalamus was analysed among the most and least aggressive fish at each density. Fish reared at high density were darker, had larger brains, were less active and less aggressive than those reared at low density and had differentially expressed genes consistent with a reactive stress‐coping style and activation of the hypothalamus–pituitary–interrenal (HPI) axis. Differences in gene expression among aggressive fish were accounted for by density and the interaction between density and aggression levels, whereas for nonaggressive fish differences in gene expression were associated with individual variation in skin brightness and social stress. Thus, the response to crowding in Nile tilapia is context dependent and involves different neuroendocrine pathways, depending on social status. Knowledge of genes associated with the response to crowding may pave the way for more efficient fish domestication, based on the selection of nonaggressive individuals with increasing tolerance to chronic stress necessary for aquaculture intensification.

## 1 INTRODUCTION

To meet global fish demand, aquaculture will need to produce more fish with less food, less water and less space in the future (Godfray et al., 2010; Goldburg & Naylor, 2005). This will require the selection of fish that can thrive under crowded conditions and adapt well to life in captivity (Huntingford, 2004; Huntingford et al., 2006) while maintaining high welfare standards (Ashley, 2007; FAWC, 2014). But to select fish that perform well at high densities requires knowledge on the genetic basis of social behaviours, which for most farmed fish is lacking. Fish domestication involves profound changes in social behaviour (Huntingford et al., 2006), but knowledge on the expression of genes underlying social behaviours has mostly focused on model or “simple” organisms (Sokolowski, 2010) or in relation to the production of terrestrial livestock (Mormède, 2005).

The genetic basis of social behaviours has historically been difficult to study (Blumstein et al., 2010), as these encompass complex phenotypic traits that often depend on genotype by environment interactions (Komers, 1997; Robinson, Fernald, & Clayton, 2008). Aggression is one of the most ubiquitous social behaviours (Maxson & Canastar, 2005), but also one of the most labile ones, as it is easily influenced by the environment experienced during early development (Fernald, 2012; Marks, West, Bagatto, Moore, & Taylor, 2005; Maruska, 2015; Trainor, Lin, Finy, Rowland, & Nelson, 2007). Individuals resort to aggressive behaviour to protect themselves and their progeny, to compete for resources and mating partners or to establish a social rank that might accrue future benefits (Nelson & Trainor, 2007). A loss of aggression is possibly the most pronounced behavioural change that accompanies animal domestication (Price, 1999), but there is controversy about the underlying molecular mechanisms (Wilkins, Wrangham, & Fitch, 2014). In cultured fish, rearing density has a marked influence on aggression, but its effects are complex and species‐specific (Huntingford, 2004; Huntingford et al., 2006). For example, a breakdown of agonistic behaviours (i.e., social behaviour associated with aggression including threats, displays, retreats, placation and/or conciliation; Barrows, 2000) is commonly observed among nonsocial fish reared at high densities, and this can be explained by a trade‐off between maintaining a social rank and effectively competing for resources (Ellis et al., 2002). Yet, captive bred fish can either be more or less aggressive than wild counterparts, depending on species (Huntingford, 2004). While crowding may increase aggression in some species, it can suppress it in others (Martins et al., 2012). Thus, understanding the molecular basis of such contrasting responses of fish to crowding is key for selecting individuals that adapt well to captivity during aquaculture intensification.

The advent of genomic tools offers new opportunities to decipher the molecular mechanisms underlying aggression and other complex behaviours affected by domestication (Robinson et al., 2008). These studies have shown that some behaviours are orchestrated by neurohormonal and gene expression regulatory networks which are largely conserved across vertebrates (Freudenberg, Carreño Gutierrez, Post, Reif, & Norton, 2016; Goodson, 2005; O'Connell & Hofmann, 2012). Yet, one outstanding challenge is to document how the environment interacts with molecular pathways to shape variation in individual behaviours (Maruska & Fernald, 2014). For example, studies in model organisms indicate that the display of aggression depends on genotype × social environment interactions, and that these drive individual variation in agonistic behaviours (Gallardo‐Pujol, Andrés‐Pueyo, & Maydeu‐Olivares, 2013; Rohde, Gaertner, Wards, Sørensen, & Mackay, 2017). In some fish, changes in social status can alter the expression of specific genes in the brain, and these can modify skin darkening (a proxy for social stress; Höglund, Balm, & Winberg, 2000), dopamine pathways (Weitekamp, Nguyen, & Hofmann, 2017) and complex behaviours (Fernald, 2012). This suggests that changes in social stress, brought about by crowding, might be reflected in changes in gene expression, which might in turn alter behaviour.

We therefore investigated how aggression, social stress and gene expression changed during aquaculture intensification in Nile tilapia (*Oreochromis niloticus*, Linnaeus, 1758), one of the oldest and most extensively farmed fish worldwide (FAO, 2016). As most cichlids, this species is structured into social hierarchies maintained by agonistic interactions in the wild (El‐Sayed, 2006), but under aquaculture conditions rearing density has a profound effect on the morphology, physiology and behaviour of tilapia (Barcellos, Nicolaiewsky, De Souza, & Lulhier, 1999; El‐Sayed, 2002; Fessehaye, Kabir, Bovenhuis, & Komen, 2006). In particular, high stocking density causes a shift from antagonistic (aggressive) to shoaling behaviour (Gall & Bakar, 1999), and more generally, from a proactive to a reactive stress‐coping style (Champneys, Castaldo, Consuegra, & Garcia de Leaniz, 2018) that has implications for welfare and disease resistance (Ellison et al., 2018). Yet, the underlying molecular mechanisms of such a dramatic behavioural shift remain largely unknown. We compared patterns of gene expression at two contrasting densities in order to disentangle the molecular pathways responsible for the behavioural changes that accompany crowding during aquaculture intensification. Our hypothesis was that rearing density would modulate the frequency of agonistic interactions, and that individuals with different aggression levels and stress‐coping styles would differ in the expression in the hypothalamus of key genes involved in the stress response.

## 2 MATERIAL AND METHODS

### 2.1 Origin and rearing of fish

A total of 360 mixed‐sex three‐week‐old Nile tilapia (*O. niloticus*, silver strain) were sourced from a commercial supplier (Fishgen Ltd) which employs communal tank spawning, typically involving four sires and 12–15 dams per spawning tank. The fish were acclimatized at a recirculation fish facility for 3 days and were then randomly distributed into nine identical 20‐L tanks (40L × 30W × 22H cm). Six tanks were stocked with 20 fry/tank (initial biomass = 0.21 g/L, final biomass = 25.5 g/L), and three tanks were stocked with 80 fry/tank (initial biomass = 0.86 g/L, final biomass = 95 g/L). These densities are commercially relevant for tilapia farming and representative of low and high densities in recirculation aquaculture systems (Conte, 2004). The higher number of replicates used for the low density treatment (*n* = 6) compared to high density (*n* = 3) was motivated by the need to mark the same number of fish for testing (see below), but tank effects were explicitly taken into consideration in the statistical analysis.

After 10 weeks of rearing, 120 fish (10 from each of the six low density tanks and 20 fish from each of the three high density tanks) were individually marked with intraperitoneal PIT tags (7 × 1.35 mm, 30 mg, Loligo Systems), returned to their original tanks and allowed to recover for 10 days before the start of the behavioural screening. Fish were fed twice daily (Skretting), progressively reducing the ration from 20% to 5% body weight per day and increasing pellet size following commercial guidelines as per feed manufacturers' recommendations. Rearing conditions and water quality were maintained within the optimal range for the species (El‐Sayed, 2006); temperature: 25–27.5°C; dissolved oxygen > 75%; photoperiod 12D:12L).

### 2.2 Behavioural screening

**Figure 1:**
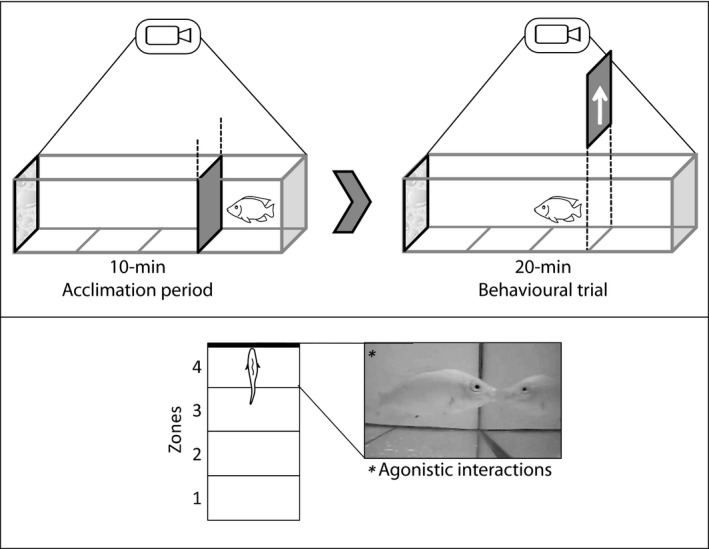
Experimental set up for mirror image stimulation used to assess aggression in Nile tilapia

We quantified the behaviour of tilapia towards their own mirror image using the mirror image stimulation (MIS) test (Balzarini, Taborsky, Wanner, Koch, & Frommen, 2014) to assess individual variation in aggression of fish reared at low and high densities. The lack of visual self‐recognition in fish supports the use of MIS to quantify aggression under repeatable conditions, unaffected by chemical cues from conspecifics (Balzarini et al., 2014; Barreto et al., 2009). Fish were assessed individually in four experimental test tanks (60L × 30W × 30H cm) consisting of an acclimation area (15L × 30W cm), fitted with a remotely operated gate door, and a mirror at the opposite end of the tank. Three lines drawn on the bottom of the tanks delimited four equal zones at varying distances from the mirror to help us assess activity levels (Figure 1). Tanks were separated by opaque walls to prevent test fish from seeing each other.

Fish were introduced singly in the acclimation zone, and after 10 min, the gate door was opened remotely and their behaviour was recorded for 20 min using two video cameras (A‐Tech Sony EFFIO 580TVL CCD Outdoor Camera) mounted above and to the side. We measured two behaviours in each fish: (a) “activity,” defined as the number of crosses between zones, and (b) “aggression,” defined by the number of agonistic actions (nips and charges) directed towards their mirror image (Barreto et al., 2009). At the end of each test, the fish were returned to their original rearing tanks, and the test tanks were washed with 90% ethanol and rinsed with distilled water to prevent the build‐up of stress hormones that might affect the behaviour of subsequent fish (Roberts, Taylor, & Garcia de Leaniz, 2011). After 10 days—to give fish time to recover from any stress associated with testing—all individually tagged fish used in the mirror test were humanely euthanized by an overdose of anaesthesia, sexed by visual inspection of the gonads and weighed. Their brains were rapidly dissected, weighed and stored in RNAlater at −20°C for subsequent analyses of gene expression.

### 2.3 Assessment of skin brightness

To assess the extent of skin brightness, photographs of each fish were taken underwater during the acclimation period using a Canon EOS 400D Digital camera and a white background fitted with a colour standard (Classic Target—X‐rite—Color Checker). Colour standardization and analysis were performed using GIMP 2.8.16 (Solomon, 2009) as per Clarke and Schluter (2011). Greyscale filtered values (0–255) of R, G and B were measured along the fish flank, between the beginning and end of the dorsal fin, and compared to the background colour standard. From this, background‐corrected average greyscale values were converted to luminance (brightness) in HSV space using the *rgbtohsv* function in the *grDevices* R base package (R Core Team, 2017).

### 2.4 Transcriptomic analysis

For transcriptomic analysis, we chose for each density the six most aggressive and six least aggressive individuals on the basis of their MIS scores (*n* = 24) and selected only males to reduce unwanted variability resulting from sex differences in gene expression (Trainor & Hofmann, 2007; Zabegalov et al., 2019). The hypothalamus of each fish was detached under a dissecting microscope; we chose this brain region because previous studies had shown it is involved in the control of social behaviours and aggression in fish (Filby, Paull, Hickmore, & Tyler, 2010; Goodson, 2005). Total RNA was extracted using the *AllPrep DNA/RNA Mini Kit* (Qiagen) following manufacturer's instructions. The final product was eluted in 40 µl RNAse‐free water. RNA quality (quantity, purity and integrity) was checked using a NanoDrop NS‐100 Spectrophotometer (NanoDrop Technologies) and an Agilent 2100 Bioanalyzer (Agilent Technologies). All RNA used for library construction was of high quality, having 260/230 and 260/230 ratios > 1.8 and RIN scores > 8. Individual cDNA libraries were prepared using Illumina TruSeq RNA sample preparation kit (1ug of total RNA; 8p.m. final concentration) and quantified using a Qubit Fluorometer (Invitrogen). The resulting 24 libraries were indexed, pooled and sequenced on an Illumina HiSeq2500 platform (2 lanes‐2 × 126 bp).

Low‐quality reads and Illumina Truseq adaptors were filtered out using Trimmomatic v 0.33 (Bolger, Lohse, & Usadel, 2014), excluding reads that were <36 bases long. After a quality check using FastQC v0.11.2 (Andrews, 2010), we mapped mRNA‐seq reads using the Tophat 2.1.1 and Cufflinks 2.2.1 (Linux ×86‐64) pipeline (Trapnell et al., 2012) to the available tilapia (*O. niloticus*) genome assembly (Orenil 1.0, accession number PRJNA5957, Brawand et al., 2014. Sequenced and assembled at the Broad Institute from a female Nile tilapia originating from a clonal line provided by D. Penman, Institute of Aquaculture, Stirling, UK). Between 15 and 20 million reads per sample were mapped to the genome, representing 87%–98% of all generated reads, and 70% of these were mapped to Ensembl annotated coding regions. Using uniquely mapped reads from Tophat, read counts per exon were obtained using the summarize overlaps function from the *Genomic Alignments* package (Lawrence et al., 2013), predefining gene models, grouping exons by gene for counting reads using Ensembl Tilapia's GTF file.

### 2.5 Statistical analyses

All statistical analyses were conducted with R 3.3.3 (R Core Team, 2017). To model the effects of rearing density on fish mass, we used a linear mixed model approach (LMM) with rearing density and sex as fixed factors and tank identity as a random factor. To achieve model simplification, we started with a full model with all main effects and interactions and selected the model with the lowest AIC value via backward selection using the *step* function in the *lmerTest* package (Kuznetsova, Brockhoff, & Christensen, 2017) which was then refitted via restricted maximum likelihood or as a linear model when the random component was not significant when compared to the fixed effects only model by the log likelihood ratio (LRT). The same approach was used to assess the effects of rearing density on brain weight and skin brightness, using rearing density, body mass and sex as predictors, and tank identity as a random effect.

To model the effect of rearing density on activity and agonistic interactions (both count data), we employed a generalized linear mixed model (GLMM) with a Poisson log‐link while statistically controlling for the effects of body mass and sex. We corrected for overdispersion using fish identity nested within tank to generate a random effect with one level per observation using the *lme4* R package (Bates, Maechler, Bolker, & Walker, 2014), as suggested by Harrison (2014). To achieve model simplification, we started with a full model with all main effects and selected the model with the lowest AICc value using the *dredge* function in the *MuMIn* R package (Barton, 2015). We used the ANOVA command to compare the null model with only a random effect grouping structure and the most plausible mixed model with both random and fixed effects.

Read counts per gene were analysed with the *DESeq2* R package, based on the negative binomial distribution (Love, Huber, & Anders, 2014). Counts were prefiltered with a threshold of >20 reads per sample in more than one sample via the independent filtering component of DeSeq2, using default parameters to optimize model sensitivity and improve computational speed (Love et al., 2014). As the number of attacks and extent of body brightness differed significantly between densities, these were also included as predictors in the analysis. Initial inspection of the data by PCA and hierarchical clustering indicated the existence of two extreme values (one from each density; Figure S1a). Although not a single standard exists for dealing with outliers in RNA‐seq analysis (Conesa et al., 2016), as these two outliers were outside two standard deviations of the median of the first two principal components they were excluded from analysis (Ellis et al., 2013). Inspection of variance inflation factors (VIF) indicated low multicollinearity for both aggressive (VIF on PC1 = 2.7, 3.2 and 5.9) and nonaggressive fish (VIF on PC1 = 1.4, 1.6 and 1.1).

After removing the two outliers and filtering low counts, 18,963 Ensembl genes were used for exploratory and differential expression analysis. PCA inspection showed no obvious clustering by density, skin brightness or aggression intensity after outlier removal (Figure S1b), but revealed a much higher variation for nonaggressive fish than for aggressive ones (Figure S2). Different intragroup variability is a well‐known problem in RNA‐seq data analysis, as it can inflate per gene dispersion estimates and reduce statistical power for detecting differentially expressed genes, which can go undetected (Landau & Liu, 2013). Therefore, as recommended for such cases within DESeq2 analysis (Love et al., 2014), we modelled gene expression separately for the two heterogenous groups (most aggressive and least aggressive fish) using density, number of attacks and skin brightness (and their interactions) as predictors. For comparisons, we also include the pooled results using aggression as a categorical variable in a 2‐way ANOVA (i.e., gene expression ~ density + aggression group + density: aggression group) as supplementary material (Tables [Link], [Link], [Link]). We used a false discovery rate (FDR) of *p* corrected values < 0.05 for differential gene expression (Ellison et al., 2018). Functional annotation and enrichment analysis, based on *Gene Ontology* terms, was performed using DAVID 6.8 (Huang et al., 2007).

## 3 RESULTS

### 3.1 Body mass

Body mass was unaffected by rearing density (*p* = 0.204) or the interaction between density and sex (*p* = 0.827), but males were 27% larger than females (LM estimate for males = 8.30, *SE* = 2.16, *t*_102 = _3.84, *p* < 0.001). The random component (tank identity) was not significant (LRT = 2.189, *df* = 1, *p* = 0.139) and was dropped from the model.

### 3.2 Brain weight

**Figure 2:**
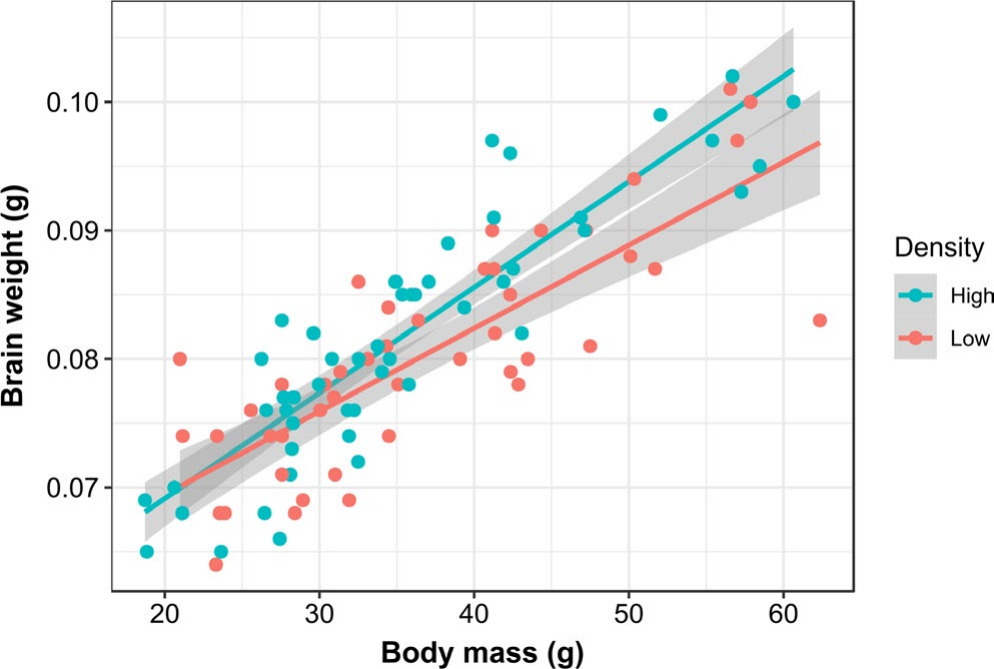
Relationship between body weight and brain weight in Nile tilapia reared at low and high density (density effect *t*_94_ = −2.54, *p* = 0.013)

Brain weight increased with body mass (*p* < 0.001) and was proportionally larger at high density than at low density (LM estimate for low density = −2.49 × e−03, *SE* = 9.82 × e−04, *t*_94_ = −2.54, *p* = 0.013, Figure 2). There were no sex differences in relative brain size (*p* = 0.722) and none of the interactions were significant (*p* > 0.5); variation among tanks (random component) was also not significant (LRT = 1.802, *df* = 1, *p* = 0.179).

### 3.3 Skin brightness

**Figure 3:**
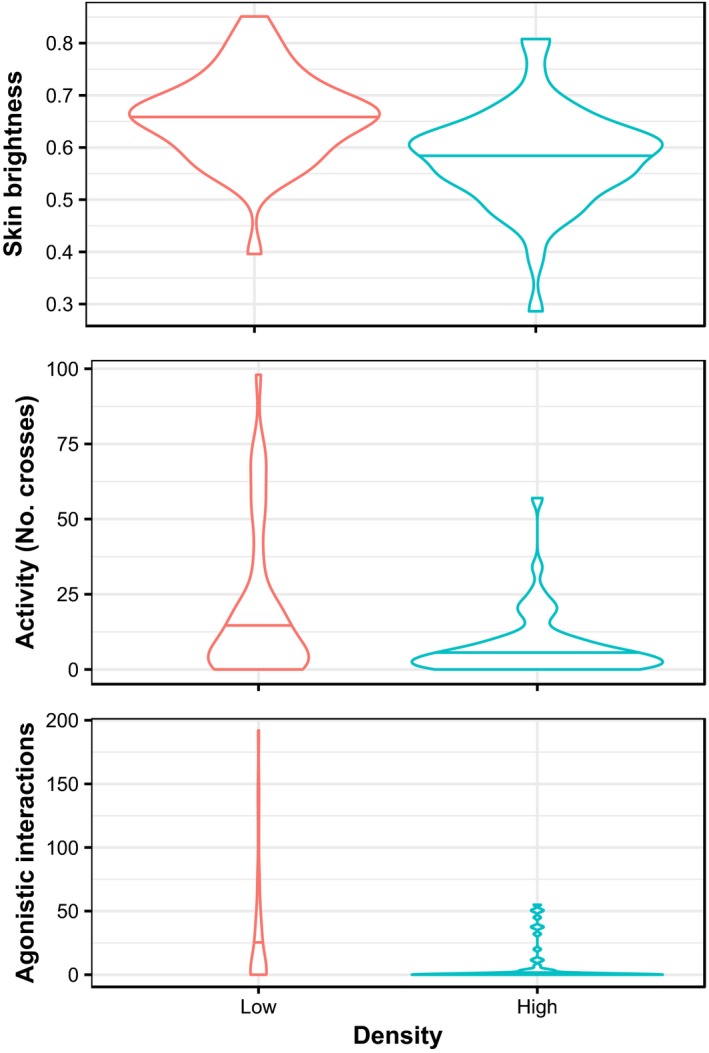
Effect of rearing density on variation of (a) skin brightness (0 = black, 1 = white), (b) activity (no. of zone crosses in the test arena) and (c) aggression (no. of agonistic interactions against a mirror image)

Tilapia reared at high density were significantly darker (i.e., had lower values of brightness) than fish at low density (LM estimate for low density = 0.072, *SE* = 0.017, *t*_105_ = 4.287, *p* < 0.001; Figure 3a). Skin brightness was unaffected by body mass (*p* = 0.609), sex (*p* = 0.454) or any of the interactions (*p* > 0.3). As for brain size, the random component (tank identity) was not significant (LRT = 2.274e−13, *df* = 1, *p* = 1.00) and was dropped from the model.

### 3.4 Activity

Activity levels, measured as number of crosses between zones, was unrelated to body mass (*p* = 0.952) or sex (*p* = 0.967), but was strongly influenced by rearing density (Figure 3b). Fish reared at low density were significantly more active (mean number of crosses = 19.55, *SE* = 3.33) than those reared at high density (mean number of crosses = 7.43, *SE* = 1.40), while statistically controlling for tanks effects (GLMM estimate for low density = 0.850, *SE* = 0.299, *z* = 2.841, *p* = 0.004).

### 3.5 Aggression

Aggression, measured as number of agonistic interactions against the mirror, was unrelated to body mass (*p* = 0.750) or sex (*p* = 0.514), but fish reared at low density were significantly more aggressive (mean number of interactions = 28.63, *SE* = 5.93) than those reared at high density (mean number of interactions = 7.11, *SE* = 2.09), while statistically controlling for tanks effects (GLMM estimate for low density = 3.103, *SE* = 0.887, *z* = 3.498, *p* < 0.001; Figure 3c).

### 3.6 Transcriptional analysis (RNA‐seq)

We examined the effects of rearing density, aggression intensity (number of attacks) and skin darkening (and their interactions) as predictors of gene expression in the brain of the most and least aggressive individuals at each density. After correcting for multiple testing, we found 25 responsive genes among the most aggressive fish and 41 genes among the least aggressive fish (Table 1), with only one gene (*fosab*) being responsive among both groups. Most of the variation in gene expression among aggressive fish was accounted for by density (eight genes) and the interaction between density and aggression intensity (19 genes), whereas for nonaggressive fish most of the variation in gene expression was associated with variation in skin brightness (38 genes).

**Table 1:** ENSMBL genes significantly influenced by density (low vs. high), aggression intensity (number of agonistic interaction), skin brightness or their interactions among the six most aggressive and six least aggressive fish at each density

Gene ID	log2 fold change	*SE*	stat	padj	Gene name	Gene description	GOTerm (BP)
Aggressive fish							
Influenced by density (*n* = 8)							
ENSONIG00000009137	−32.47	6.21	−5.23	0.00	krt5	keratin 5	
ENSONIG00000001057	−11.64	2.70	−4.31	0.03	sst1.1	somatostatin 1, tandem duplicate 1	0010469
ENSONIG00000002652	−9.19	2.17	−4.23	0.04	jarid2b	jumonji, AT‐rich interactive domain 2b	0006355;0007275;0016569;0031061 0051574
ENSONIG00000010340	8.47	1.92	4.40	0.03	arhgef1a	Rho guanine nucleotide exchange factor (GEF) 1a	0035556;0035023
ENSONIG00000003670	14.19	2.58	5.50	0.00	nnt	nicotinamide nucleotide transhydrogenase	0015992;0055114
ENSONIG00000019114	19.53	4.51	4.33	0.03	MFAP4 (one of many)	Microfibril‐associated protein 4	
ENSONIG00000020827	25.72	5.67	4.54	0.02	_	_	
ENSONIG00000015953	35.47	4.91	7.22	0.00	bag6l	
Influenced by number of attacks (*n* = 2)							
ENSONIG00000000379	0.13	0.03	4.58	0.04	minpp1a	multiple inositol‐polyphosphate phosphatase 1a	
ENSONIG00000015953	0.41	0.06	6.67	0.00	bag6l	BCL2‐associated athanogene 6 like	
Influenced by brightness (*n* = 1)							
ENSONIG00000015953	38.16	5.95	6.41	0.00	bag6l	BCL2‐associated athanogene 6, like	
Influenced by density × no. of attacks (*n* = 19)							
ENSONIG00000003670	0.08	0.01	7.17	0.00	Nnt	nicotinamide nucleotide transhydrogenase	0015992;0055114
ENSONIG00000004830	−0.09	0.02	−4.11	0.04	si:ch73‐335l21.4	si:ch73‐335l21.4	
ENSONIG00000006273	−0.05	0.01	−5.01	0.00	tuft1a	tuftelin 1a	
ENSONIG00000006744	−0.15	0.03	−5.61	0.00	zgc:162944	zgc:162944	
ENSONIG00000006747	−0.15	0.03	−5.68	0.00	irg1	immunoresponsive 1 homolog (mouse)	0006954;0002376;0045087
ENSONIG00000007532	−0.06	0.01	−5.45	0.00	zgc:122979	zgc:122979	0006457
ENSONIG00000009137	−0.15	0.03	−4.86	0.00	krt5	keratin 5	
ENSONIG00000009460	−0.16	0.04	−4.09	0.04	_	_	
ENSONIG00000009551	−0.06	0.01	−4.10	0.04	SPIDR	scaffolding protein involved in DNA repair	0000724;0006974
ENSONIG00000010366	−0.05	0.01	−4.89	0.00	Txnipa	thioredoxin‐interacting protein a	
ENSONIG00000012279	−0.11	0.03	−4.18	0.03	zmp:0000000801	zmp:0000000801	0007186
ENSONIG00000015140	−0.13	0.03	−4.85	0.00	_	_	
ENSONIG00000015264	−0.06	0.01	−4.49	0.01	Fosab	v‐fos FBJ murine osteosarcoma viral oncogene homolog Ab	0033555;0006355
ENSONIG00000015953	0.13	0.02	6.50	0.00	bag6l	BCL2‐associated athanogene 6, like	
ENSONIG00000016087	−0.06	0.01	−5.62	0.00	ddit4	DNA‐damage‐inducible transcript 4	0009968
ENSONIG00000016884	−0.05	0.01	−4.98	0.00	nt5dc2	5′‐nucleotidase domain containing 2	
ENSONIG00000018737	0.05	0.01	4.05	0.05	Pomca	proopiomelanocortin a	0007218;0032400;0033555
ENSONIG00000019777	−0.09	0.02	−4.21	0.03	hbae5	haemoglobin, alpha embryonic 5	0015671
ENSONIG00000021429	−0.08	0.02	−4.62	0.01	Cebpd	CCAAT/enhancer‐binding protein (C/EBP), delta	0006355
Influenced by density × brightness (*n* = 3)							
ENSONIG00000003670	19.59	3.48	5.63	0.00	Nnt	nicotinamide nucleotide transhydrogenase	0015992;0055114
ENSONIG00000010340	12.50	2.59	4.83	0.01	arhgef1a	Rho guanine nucleotide exchange factor (GEF) 1a	0035556;0035023
ENSONIG00000015953	35.63	6.16	5.78	0.00	bag6l	BCL2‐associated athanogene 6, like	
Influenced by no. of attacks × brightness (*n* = 5)							
ENSONIG00000006744	0.69	0.14	4.78	0.01	zgc:162944	zgc:162944	
ENSONIG00000006747	0.70	0.14	4.85	0.01	irg1	immunoresponsive 1 homolog (mouse)	0006954;0002376;0045087
ENSONIG00000009137	0.69	0.15	4.45	0.03	krt5	keratin 5	
ENSONIG00000015140	0.71	0.14	4.93	0.01	_	_	
ENSONIG00000015953	−0.76	0.11	−6.84	0.00	bag6l	BCL2‐associated athanogene 6, like	
Nonaggressive fish							
Influenced by density (*n* = 3)							
ENSONIG00000006467	−24.58	3.94	−6.25	0.00			
ENSONIG00000006469	−27.49	5.91	−4.65	0.02	prl	prolactin	0010469
ENSONIG00000015264	7.48	1.56	4.80	0.01	fosab	v‐fos FBJ murine osteosarcoma viral oncogene homolog Ab	0033555;0006355
Influenced by brightness (*n* = 38)							
ENSONIG00000002382	−37.14	9.17	−4.05	0.04	_	_	
ENSONIG00000015235	−24.84	3.79	−6.55	0.00	oxt	oxytocin	0050801
ENSONIG00000015218	−18.10	4.39	−4.12	0.03	avp	arginine vasopressin	0007165;0010469
ENSONIG00000003962	−17.98	3.15	−5.70	0.00	_	_	
ENSONIG00000002871	−16.93	3.24	−5.23	0.00	prdm12b	PR domain containing 12b	0006351;0006355; 0021521
ENSONIG00000007689	−13.67	2.75	−4.97	0.00	grp	gastrin‐releasing peptide(grp)	0007218
ENSONIG00000020662	−12.86	2.94	−4.37	0.01	_	_	
ENSONIG00000005071	−12.73	2.92	−4.37	0.01	_	_	
ENSONIG00000002896	−12.49	3.13	−3.99	0.04	sp8b	sp8 transcription factor b	0006351;0006355;0030326
ENSONIG00000005998	−12.16	3.08	−3.95	0.04	sytl3	synaptotagmin‐like 3	0006886;0006887; 0006906
ENSONIG00000004333	−10.17	1.93	−5.27	0.00	_	_	
ENSONIG00000001128	−9.62	1.96	−4.91	0.00	hcrt	hypocretin (orexin) neuropeptide precursor	0007218;0007631;0033555
ENSONIG00000009224	−9.23	1.88	−4.91	0.00	_	_	
ENSONIG00000010719	−8.92	2.23	−4.00	0.04	si:ch211‐236k19.2	si:ch211‐236k19.2	0006355
ENSONIG00000004631	−8.80	2.11	−4.18	0.03	col8a2	collagen, type VIII, alpha 2	
ENSONIG00000014386	−7.94	1.46	−5.44	0.00	arxa	aristaless‐related homeobox a	0003322;0006351;0006355
ENSONIG00000019324	−7.58	1.91	−3.96	0.04	tprg1	tumour protein p63 regulated 1	
ENSONIG00000003653	−7.40	1.53	−4.84	0.00	_	_	
ENSONIG00000001403	−7.31	1.86	−3.93	0.04	meis2a	Meis homeobox 2a	0006355;0048703;
ENSONIG00000003843	−5.75	1.08	−5.31	0.00	nts	neurotensin	0010469
ENSONIG00000017229	−4.67	1.18	−3.95	0.04	trh	thyrotropin‐releasing hormone	0001692;0007165;0009755
ENSONIG00000014561	−4.50	1.01	−4.48	0.01	tacr3a	neuromedin‐K receptor‐like	0007165;0007186
ENSONIG00000019723	−4.20	1.04	−4.05	0.04	s100a10a	S100 calcium‐binding protein A10a	0042127
ENSONIG00000009148	−3.64	0.86	−4.22	0.02	vax1	ventral anterior homeobox 1	0006351;0006355;0007275
ENSONIG00000016412	−2.88	0.65	−4.45	0.01	_	_	
ENSONIG00000007099	−2.73	0.66	−4.13	0.03	_	_	
ENSONIG00000014947	−2.68	0.67	−3.99	0.04	CACNA2D1	calcium voltage‐gated channel auxiliary subunit alpha2delta 1	1904646;0070588
ENSONIG00000016069	−2.59	0.52	−5.02	0.00	_	_	
ENSONIG00000008322	−2.58	0.64	−4.05	0.04	dhrs13a.3	dehydrogenase/reductase (SDR family) member 13a, duplicate 3	
ENSONIG00000000606	2.10	0.54	3.86	0.05	eif4a1a	eukaryotic translation initiation factor 4A1A	0006412;0006446;0016926
ENSONIG00000014333	2.27	0.56	4.03	0.04	syn2b	synapsin IIb	0007269
ENSONIG00000006168	2.69	0.67	3.99	0.04	_	_	
ENSONIG00000010283	3.36	0.77	4.39	0.01			
ENSONIG00000016676	4.54	1.14	3.98	0.04			
ENSONIG00000009325	4.69	1.20	3.91	0.05	CYR61	cysteine‐rich angiogenic inducer 61	0001558;0007155;0007165; 0007267;0060548
Influenced by density × brightness (*n* = 1)							
ENSONIG00000006467	−38.04	6.79	−5.60	0.00	_	_	

Note

Positive log2 fold change values denote downregulation at high density (HD) compared to low density (LD), while for continuous predictors they represent rate of unit change.

**Figure 4:**
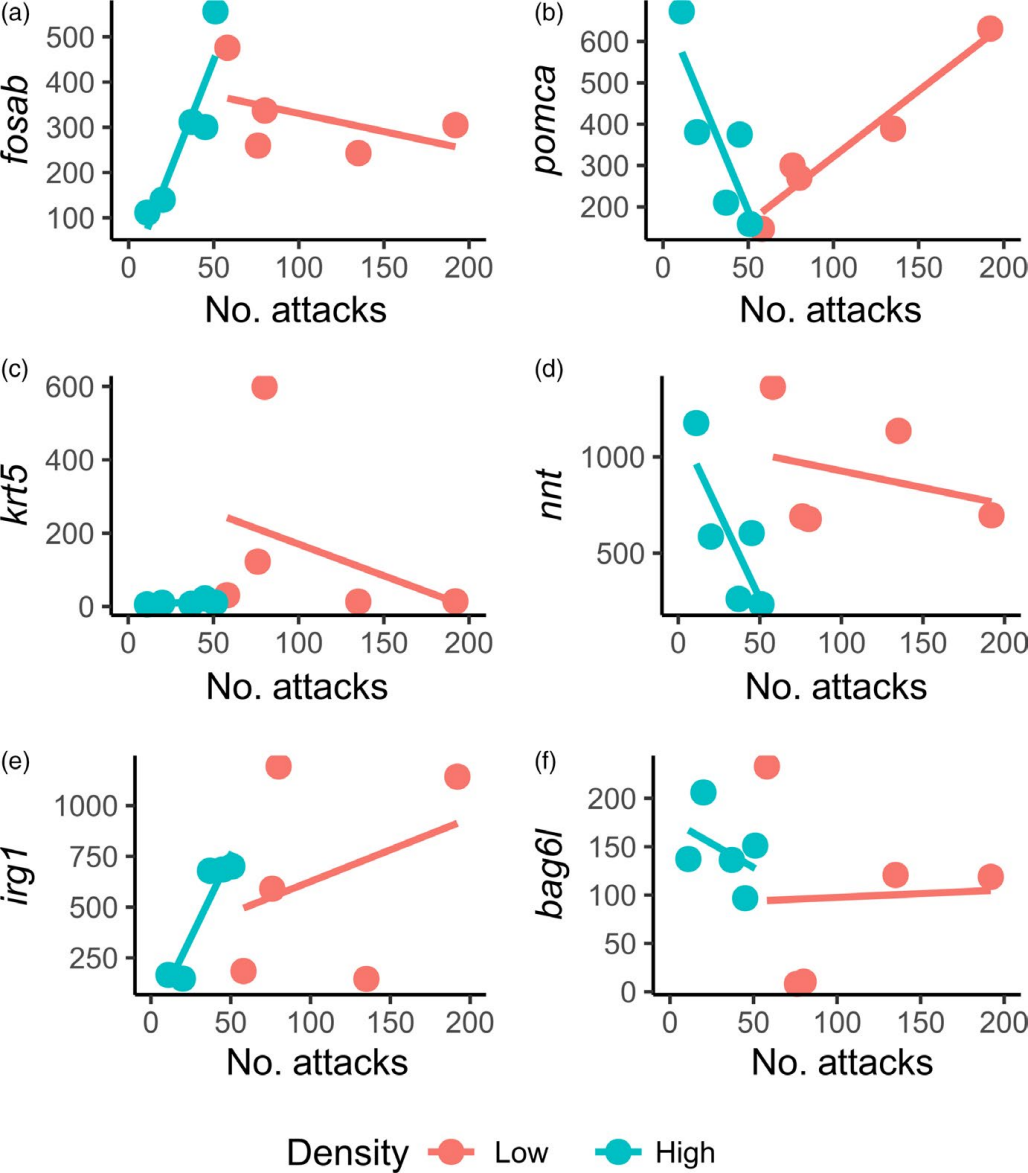
Relationship between aggression intensity (no. of agonistic interactions) and gene expression (DESeq2 normalized counts) in the six most aggressive fish at each density. Shown are examples of six responsive genes (out of 19) where there were significant interactions between aggression intensity and rearing density (*q* < 0.05; Table 1), suggesting that agonistic behaviour is regulated in a density‐dependent manner: (a) v‐fos FBJ murine osteosarcoma viral oncogene homolog Ab (*fosab*), (b) proopiomelanocortin a (*pomca*), (c) *krt5*, (d) *nnt*, (e) *irg1* and (f) *bag6l*

The eight genes that were differentially expressed between high and low density among aggressive fish included somatostatin (*sst1*), a gene that encodes for a hormone involved in numerous cellular process, *jarid2b—*involved in transcription and histone methylation regulation, *krt5—*involved in the immune response and *bag6l—*involved in apoptosis and cellular stress. The expression of 19 genes showed significant aggression × density interactions, including genes involved in organismal response to stress such as v‐fos FBJ murine osteosarcoma viral oncogene homolog Ab (*fosab; q* = 0.010; Figure 4a), proopiomelanocortin (*pomca*)—a gene that encodes a peptide hormone (ACTH) involved in the release of cortisol (*q* = 0.048; Figure 4b), *krt5* (skin stress; *q* < 0.01; Figure 4c), *nnt* (mitochondrial antioxidant defence; *q* < 0.01; Figure 4d), *irg1* (inflammation process and response to bacterial infections; *q* < 0.01; Figure 4e) and *bag6l* (apoptosis and cellular stress; *q* < 0.01; Figure 4f). Other significant interactions in gene expression included density × skin brightness (three genes) and number of attacks × skin brightness (five genes, Table 1).

**Figure 5:**
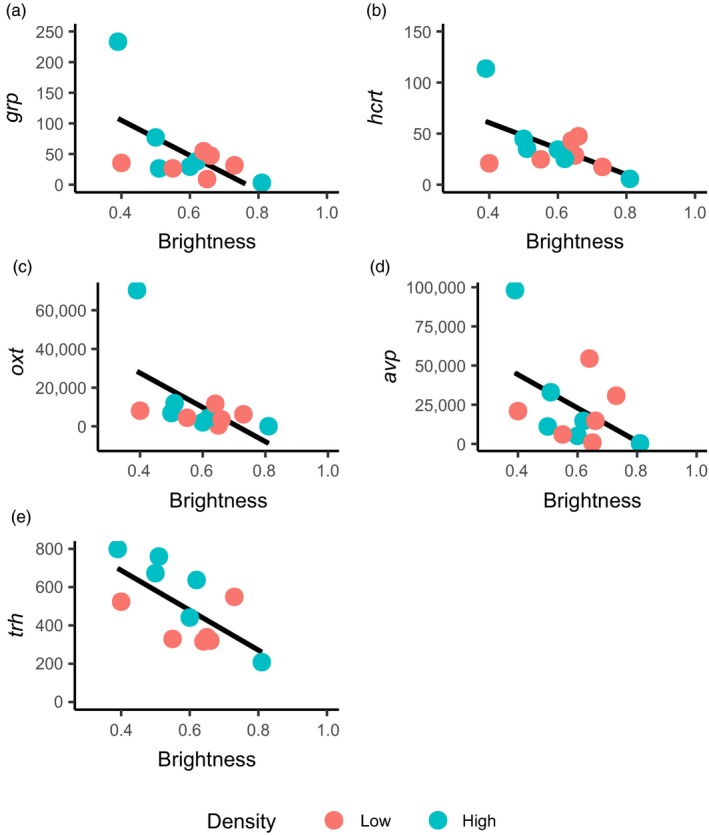
Relationship between skin brightness (a proxy for stress) and gene expression (DESeq2 normalized counts) in the six least aggressive fish at each density. Shown are examples of five responsive genes (out of 38) where there was a significant negative relationship between skin brightness and gene expression (*q* < 0.05; Table 1) independently of rearing density, suggesting a common stress response: (a) *grp*, (b) *hcrt*, (c) oxytocin (*oxt*), (d) arginine vasopressin (*avp*) and (e) thyrotropin‐releasing hormone (*trh*)

Among nonaggressive fish, 38 genes were significantly associated with skin brightness, being in most cases upregulated among dark fish and downregulated among pale ones (Table 1). These included genes involved in the stress response and ion homeostasis (*oxt, avp*), neuropeptides involved in feeding behaviour (*grp, hcrt*), and several genes that coded for hormone receptors, including the thyrotropin‐releasing hormone (*trh*) and neurotensin (*nts*) involved in dopamine signalling, as well as homebox transcription factors (Figure 5). Three genes were differentially expressed between rearing densities, including prolactin (*prl*) and *fosab* which were also affected by the interaction between density and brightness (*q* = 0.078), as well as one uncharacterized gene (Table 1).

## 4 DISCUSSION

Fish domestication is thought to be constrained by the capacity of individuals to adapt to high densities in captivity (Ashley, 2007; Huntingford & Adams, 2005), and our study shows that one consequence of crowding in Nile tilapia is the inhibition of aggression, a process that we found was associated with the expression in the hypothalamus of stress‐related genes. Stress has a profound effect on the hypothalamic–pituitary–interrenal (HPI) axis and the neuroendocrine response of zebrafish (Pavlidis, Sundvik, Chen, & Panula, 2011; Pavlidis, Theodoridi, & Tsalafouta, 2015), but the transcriptional response of farmed fish to the increase in density that accompanies aquaculture intensification is not well understood. We employed a mirror test to quantify aggression and skin darkening (a proxy for stress) at low and high density and screened the most and least aggressive individuals at each density for differential gene expression in the hypothalamus in order to examine the transcriptional response to crowding. We found that tilapia reared at high density were darker, less active and less aggressive than fish reared at low density, and that these differences were associated with transcriptional differences in the brain.

Rearing density affects the nature and strength of social interactions in many fish (Ashley, 2007) and in species such as tilapia, where social hierarchies are maintained by agonistic interactions, aggressive behaviour can be used as a predictor of stress (Barreto et al., 2009). Our results suggest the existence of two types of stress in tilapia: social stress caused by the formation of social hierarchies maintained by agonistic interactions at low density and chronic stress caused by crowding at high density.

Although only a relatively small number of genes were differentially expressed in our study, this is common in studies of gene expression in the brain and can be explained by the wide range of specialized neuronal cell types present in the brain, and in the hypothalamus in particular (Machluf, Gutnick, & Levkowitz, 2011), and the tight homeostatic balance of the nervous system (Aubin‐Horth, Landry, Letcher, & Hofmann, 2005; Filby et al., 2010). Our results are also consistent with those reported for zebrafish, where 70 genes were differentially expressed in the brain of fighting and nonfighting (isolated) individuals (Malki et al., 2016), seven of which were homologous to those differentially expressed in aggressive and nonaggressive mice.

Of the various genes that were differentially expressed between high and low density among aggressive individuals, somatostatin (*sst1*) has previously been linked to aggression in fish (Filby et al., 2010; Trainor & Hofmann, 2006), but the link is complex and appears to be species‐specific. Thus, while upregulation of *sst* was found to inhibit aggression in the African cichlid *Astatotilapia burtoni* (Trainor & Hofmann, 2006), a species that switches from territorial to nonterritorial depending on the social environment, in zebrafish, a typically shoaling fish, the opposite appears to be true (Filby et al., 2010). In our study, *sst1* was upregulated among fish reared at high density, which were significantly less aggressive than those reared at low density, suggesting an inhibitory role for somatostatin on aggression in Nile tilapia, as seen in other cichlids (Hofmann & Fernald, 2000; Trainor & Hofmann, 2006, 2007). Thus, *sst1* appears to be one of the key genes regulating the different response of species to crowding. The two other density‐dependent genes differently expressed among aggressive fish, *pomca* and *fosab*, are both involved in the stress response (Eissa & Wang, 2016), and their expression in our study depended on the interaction between rearing density and aggression. We found a negative association between *pomca* expression and number of attacks at high density, but a positive association at low density. As *pomca* encodes a preproprotein whose proteolytic products include adrenocorticotropin (ACTH), which stimulates cortisol secretion (Bornstein & Chrousos, 1999), this suggests that elevated levels of aggression may result in increased cortisol production. Indeed, a recent study in a group living cichlid has shown that aggressive behaviour is associated with higher production of cortisol (Culbert, Gilmour, & Balshine, 2018). Another proteolytic product of *pomca* includes the alpha‐melanocyte‐stimulating hormone, α‐MSH, which disperses pigment‐containing melanosomes in the pigment cells, thereby making fish look darker (Kobayashi, Mizusawa, Chiba, Tagawa, & Takahashi, 2012; Kobayashi, Mizusawa, Saito, & Takahashi, 2012). We found that body darkening (i.e., lower brightness) was significantly associated with high density, adding support to the idea that body darkening is a good proxy for chronic stress in Nile tilapia (Champneys et al., 2018), as seen in other fish (Höglund et al., 2000). In zebrafish, aggressive individuals are also darker, while those that exhibit fear become paler (Gerlai, Lahav, Guo, & Rosenthal, 2000), which serves to highlight the relationship that exists between social stress, skin darkening and aggression.

An interaction between rearing density and aggression was also found with respect to the expression of *fosab* among aggressive fish. We found that *fosab* expression was positively associated with aggression intensity at high density, but negatively associated at low density. *Fosab* encodes c‐Fos, whose upregulation has previously been associated to both stress (Kovács, 1998; Pavlidis et al., 2015) and aggression (Davis & Marler, 2004; Haller, Tóth, Halasz, & De Boer, 2006; Malki et al., 2016), and which also modulates neural plasticity underlining behavioural flexibility (Oliveira, 2012). This suggests that expression of *fosab* and aggression in tilapia are context dependent and are driven by social stress caused by agonistic interactions at low density and by crowding and chronic stress at high density. Aggression is influenced by genotype × environment interactions, notably social stress, in mammals and fish alike (Zabegalov et al., 2019), and many genes that have been associated with aggression and stress in mammalian studies respond in a similar manner in zebrafish (Freudenberg et al., 2016). These include *oxt*, *avp*, *hcrt* and *sst1* which are upregulated in dominant zebrafish, and which we found in our study to be influenced by skin darkening (and hence social stress) and density (*sst1*) in Nile tilapia.

Crowding has previously been found to have a darkening effect on fish, usually associated with increased chronic stress (Brown & Shahidi, 1997; Van der Salm, Martinez, Flik, & Wendelaar Bonga, 2004; Zeng et al., 2010). Among nonaggressive fish, we found a negative association between brightness and the expression in the hypothalamus of several important neuropeptides, hormones and hormone receptors, including the gastrin‐releasing peptide (*grp*), hypocretin/orexin neuropeptide precursor (*hcrt*), oxytocin (*oxt*), vasopressin (*avp*), thyrotropin‐releasing hormone (*trh*), parathyroid hormone 1 receptor (pth1ra) and the growth hormone‐releasing hormone receptor (*ghrhrl*). All these genes have previously been reported to be upregulated in stressed or subordinate fish (Balment, Lu, Weybourne, & Warne, 2006; Bernier, 2006; Jezova, Skultetyova, Tokarev, Bakos, & Vigas, 1995; Pavlidis et al., 2011, 2015). The expression of prolactin (*prl*) and *fosab* also depended on the interaction between density and body darkening, though the effect was not strong. Prolactin is involved in the regulation of body pigmentation (Leclercq, Taylor, & Migaud, 2010; Oshima, Makino, Iwamuro, & Bern, 1996), and a common response to stress is an increase in prolactin secretion and mRNA levels (Auperin, Baroiller, Ricordel, Fostier, & Prunet, 1997; Avella, Schreck, & Prunet, 1991; Pavlidis et al., 2015), and hence darkening. Upregulation of *fosab* has also been associated with an increase in stress and skin darkening in other species (Kovács, 1998; Pavlidis et al., 2015), suggesting that both prolactin and *fosab* are conserved indicators of chronic stress caused by crowding.

The response of fish to stress depends not only on the type of stressor but also on the coping style that characterizes an individual (Koolhaas, De Boer, Coppens, & Buwalda, 2010; Koolhaas et al., 1999). Thus, the response along the HPI axis can be viewed as a dynamic multivariate system, where the effect of each element depends on the intensity of the others (Pavlidis et al., 2011, 2015). In our study, and with one exception (*fosab*), aggressive and nonaggressive fish expressed different genes in relation to rearing density, and among aggressive fish there were significant density‐dependent interactions between aggression intensity and gene expression, suggesting that different neuroendocrine pathways may be involved. The existence of significant interactions between rearing density and aggression, and between density and body darkening, serves to highlight the fact that the regulation of aggression not only varies among individuals, but is also context dependent and shaped by social stress. On the other hand, aggression is one of the most repeatable and heritable fish behaviours (Bell, Hankison, & Laskowski, 2009; Way, Ruhl, Snekser, Kiesel, & McRobert, 2015), which might facilitate the selective breeding of nonaggressive individuals with high tolerance to stress under crowded conditions. For example, several serotonergic genes and genes related to dopamine signalling and to c‐Fos found in our study are also differentially expressed between aggressive and nonaggressive in zebrafish (Malki et al., 2016; Zabegalov et al., 2019), suggesting that these could be used as biomarkers for aggression. In addition, there is mounting evidence for an epigenetic regulation of aggression in humans and rodents (Waltes, Chiocchetti, & Freitag, 2016), which given the conserved nature of many cellular networks implicated in aggression across taxa (Malki et al., 2016), might also make it possible to use epigenetic conditioning to reduce aggression and stress under aquaculture intensification, as shown recently for disease resistance (Uren Webster et al., 2018).

Beyond the observed transcriptional and behavioural changes associated with crowding, tilapia reared at low density had smaller brains (relative to their body size) than fish reared at high density. In mammalian systems, chronic stress can impact on brain structures through the production of glucocorticoids which can in turn affect cognition and social interactions (Lupien, McEwen, Gunnar, & Heim, 2009), and it is possible that stress has a similar effect on the brain size of fishes. Fish brain size can evolve very rapidly in response to environmental conditions, reflecting a trade‐off between enhanced cognition and reproductive performance (Kotrschal et al., 2013). For example, fish living in the wild develop larger brains compared to hatchery‐reared conspecifics living in more impoverished environments (Kihslinger, Lema, & Nevitt, 2006), as do fish living in high predation areas compared to habitats with fewer predators (Kotrschal, Deacon, Magurran, & Kolm, 2017). This suggests that the smaller brain of fish reared at low density may have been the consequence of social stress caused by elevated aggression, and perhaps more generally, a trait associated with a proactive (rather than a reactive) stress‐coping style (Champneys et al., 2018).

## 5 CONCLUSIONS

Our study indicates the crowding inhibits aggressive behaviour in Nile tilapia and results in changes in the expression of stress‐related genes that accompany the shift from social hierarchies maintained by agonistic interactions at low density, to shoaling at high density. This suggests that aquaculture can substantially alter the aggression level and stress response of Nile tilapia. Given that loss of aggression and stress tolerance are two of the defining features of animal domestication (Belyaev, 1969; Jensen, 2014), and that our study shows that these were associated with differential gene expression in Nile tilapia, it might be possible to selectively breed fish that perform well under crowded conditions under aquaculture intensification. Some of the genes identified here, such as *sstl* and *fosab*, will be key candidates for this, as they seem to play an important role in the responses of different species to crowding stress.

## CONFLICT OF INTEREST

The authors declare no conflict of interest.

## AUTHOR CONTRIBUTIONS

SC and CGL wrote the grant and secured the funding. OR, CGL and SC designed the study. OR, DRB, GC and TUW collected the data. CGL, DRB and OR wrote the MS with input from all authors. DRB and CGL carried out the statistical analysis. All authors approved the final submission.

## ANIMAL ETHICS

This work was screened and approved by Swansea University's College of Science Ethics Committee (permit BS 09062016).

## Data Availability

All sequences have been submitted to NCBI SRA (Submission ID: SUB4925804; BioProject: PRJNA510732; BioSample SAMN10614327‐SAMN10614330). Phenotypic and behavioural data are available from Figshare (https://doi.org/10.6084/m9.figshare.8198408.v1) Rodriguez‐Barreto et al., 2019
