# Supplementary material for: Transcriptomic response to aquaculture intensification in Nile tilapia
Source: Evol Appl. 2019 Jul 17;12(9):1757–71. doi: 10.1111/eva.12830 (PMC6752142; doi:10.1111/eva.12830)
Supplement: Supplementary file 1 [file EVA-12-1757-s001.docx]

**Figure S1.** (A) PCA of all samples (n = 24) showing the existence of two potential outliers with values outside two 2SD from the median (fish A05-18, PC1 = 27.01, PC2 = 14.60; fish A01-06, PC1 = 15.44, PC2 = 21.31; 2SD PC1= 16.02; 2SD PC2 = 13.45). (B) PCA after outlier removal (n=22).


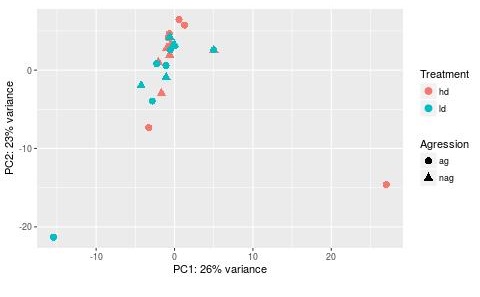


outliers

**Density**

**Aggression Density**

High

Low

Aggressive

Non-aggressive

**A**


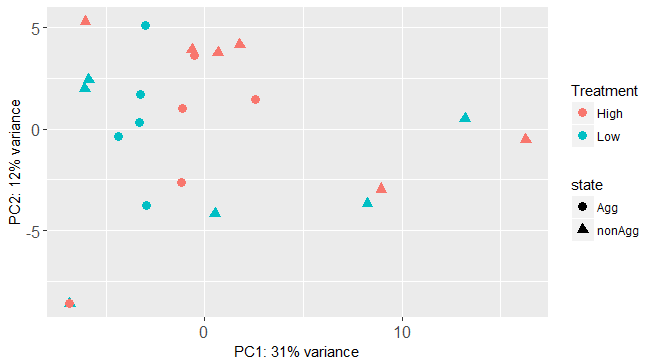


**B**
