# Supplementary material for: Transcriptomic response to aquaculture intensification in Nile tilapia
Source: Evol Appl. 2019 Jul 17;12(9):1757–71. doi: 10.1111/eva.12830 (PMC6752142; doi:10.1111/eva.12830)
Supplement: Supplementary file 2 [file EVA-12-1757-s002.docx]

**Figure S2**. Within group variation in gene expression along PC1 of aggressive and non-aggressive Nile tilapia reared at high and low density.
